# Soft Probe Electrical Contact: Eliminating Electrode Deposition and Enabling Reliable Measurements of Emerging Materials

**DOI:** 10.3390/ma19132738

**Published:** 2026-06-26

**Authors:** Michiko Yoshitake, Kentaro Kinoshita, Hiroki Matsuo, Seiji Sakai, Songtian Li

**Affiliations:** 1 National Institute for Materials Science, Tsukuba 305-0047, Japan; 2 Faculty of Advanced Engineering, Tokyo University of Science, Katsushika-ku, Tokyo 125-8585, Japan; kkinosita@rs.tus.ac.jp; 3 Faculty of Advanced Science and Technology, Kumamoto University, Kumamoto 860-8555, Japan; matsuo_h@cs.kumamoto-u.ac.jp; 4 National Institutes for Quantum Science and Technology, Takasaki 370-1292, Japan; sakai.seiji@qst.go.jp (S.S.); li.songtian@qst.go.jp (S.L.)

**Keywords:** robust electric contact, vibration resistance, thermal stress resistance, electrode-less, non-destructive

## Abstract

**Highlights:**

- Electrical contact without force control was successfully demonstrated in practical applications.
- Damage-free electrical contact to MOF specimens during temperature change.
- Reliable hysteresis measurements of ferroelectric materials.
- Stable electrical measurement under environments involving vibration and thermal stress.

**Abstract:**

Electrical measurements of emerging materials such as thin films, two-dimensional materials, and fragile porous systems are often hindered by damage and contamination caused by conventional contact methods, including metal electrode deposition. In this study, we demonstrate the novelty and advantages of a mechanically compliant “soft probe” over conventional methods and conductive AFM. The non-destructive soft probe achieves stable electrical contact in the repulsive-force regime using a hairpin-shaped spring structure, allowing consistent measurements without active force control nor electrode fabrication. Case studies demonstrate that the soft probe prevents metal penetration and preserves intrinsic properties, as demonstrated in NiO resistive switching devices, and improves interface quality compared to deposited electrodes in ferroelectric measurements. It also enables electrical characterization of fragile materials such as metal–organic frameworks without inducing structural degradation. Furthermore, its mechanical compliance ensures stable operation under vibration and thermal stress, enabling measurements in vacuum and low-temperature environments. These results indicate that the soft probe provides a simple, versatile, and contamination-free platform for reliable electrical measurements, and represents a promising approach for the characterization of a wide range of emerging material systems.

## 1. Introduction

In recent years, various non-traditional materials, such as two-dimensional (2D) materials [1,2,3] and metal–organic frameworks (MOFs) [4,5,6] have emerged and attracted significant attention for a wide range of applications. Electrical measurements are essential both for the fundamental characterization of newly discovered materials and for the implementation of emerging materials in device applications [7,8,9,10,11,12,13,14,15,16,17]. In many electrical measurement systems, commercial probe stations equipped with metal needle probes are commonly used; however, these probes often scratch specimens and damage their surfaces. To address this issue, metal electrode evaporation [7,18,19] or conductive atomic force microscopy (AFM) [20,21,22,23] has typically been employed to establish electrical contact with specimens. However, these approaches have several drawbacks. Metal evaporation can contaminate the specimen, thereby preventing subsequent measurements, while AFM-based techniques require precise feedback control and complex operation. In addition, some materials are susceptible to thermal damage during metal evaporation [24,25,26,27], and therefore cooling during evaporation is often necessary [28]. Furthermore, the penetration of evaporated metal into organic layers poses a serious problem [29,30,31,32]. To overcome these limitations, we have developed a specially designed contact probe that enables robust and non-destructive electrical contact with nanometer-thick films, 2D materials, and fragile specimens without introducing contamination from electrode metals [33,34]. This probe is referred to as a “soft probe”. The developed soft probe is now commercially available in various lengths, allowing control of the applied contact force, and can be directly connected to standard coaxial cables used in measurement systems [35,36].

In addition to enabling non-destructive and robust electrical contact, the soft probe offers several practical advantages that have been recognized by users. Although some measurement data for ReRAM and MOF specimens were partly shown in our prior work [37], these advantages have not yet been systematically demonstrated with concrete experimental examples or accompanied by clear physical explanations of their origin. Therefore, this study focuses on elucidating these advantages, namely, “prevention of electrode-metal penetration”, “elimination of electrode deposition for ferroelectric hysteresis measurements”, “applicability to small and fragile materials”, and “vibration- and thermal stress-resistant measurements”, through representative case studies, including previously reported examples. By providing physical interpretations of the observed behaviors and defining the applicable range of the soft probe based on these insights, this article aims to help readers and potential users assess whether the soft probe can solve electrical-contact problems in their own measurements. It should be noted that this article concerns only electrical contact. Details of the specimens and analyses of the measurement results are provided in the references authored by the individual co-authors.

## 2. Principles of the Soft Probe

**Figure 1:**
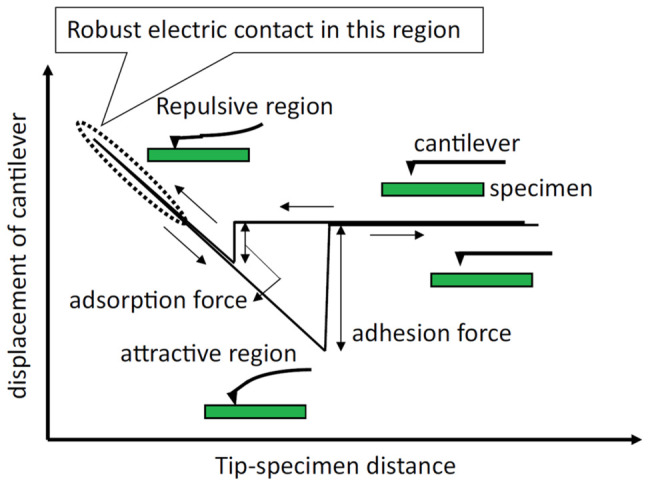
Schematic force curve in AFM showing region for robust electrical contact (cited from previous paper [37]). Arrows are the trace of cantilever approach and retract.

The principle of the non-destructive yet robust electrical contact is based on the realization of a repulsive-force regime, analogous to non-destructive AFM (see Figure 1), but implemented at the micrometer scale without active force control. The underlying principles have been described in detail elsewhere [33,37]. The key concept is that stable contact within the repulsive force regime can be achieved through direct visual observation, without the need for feedback control. In this system, the observable deflection of the probe is on the order of 1 mm [37], enabling practical manual operation. The deflection δ of a cantilever with a circular cross-section and a hairpin shaped geometry can be expressed as

$$\delta =\frac{32Pl^{3}}{3\pi Ed^{4}}$$

where *P* is the applied load (i.e., the contact force), *d* is the diameter of the wire, *l* is the length of the wire, and *E* is the Young’s modulus of the wire material [38]. Based on AFM force–distance measurements for Cu, the contact pressure corresponding to the non-destructive repulsive force regime is estimated to be approximately 5 × 10^7^ Pa. The applied load *P* can therefore be approximated as the product of this pressure and the contact area, which is roughly estimated as 0.5 × 10^−8^ m^2^. This contact area corresponds to a circular region with a radius on the order of 10 μm, as estimated for a contacting Au hemisphere with a diameter of 700 μm and a Young’s modulus of 80 GPa. Practical operation of the contact probe involves bringing the specimen toward the probe until the support wire bends by approximately 1 mm and then maintaining the sample-probe position.

It should be noted that the force-deflection curve is independent of the sample material, because it is determined by the spring constant of the probe (the hairpin shaped cantilever). By rearranging the above equation, the spring constant *κ* is given by the following equation:

$$\kappa =\frac{3\pi Ed^{4}}{32l^{3}}.$$

The yield stress (irreversible deformation threshold) of materials is on the order of several tens of MPa for both metals and plastics [39,40]. Therefore, the contact pressure should be kept below the yield stress to ensure non-destructive contact. By appropriately selecting the geometric parameters *l* and *d* such that the deflection is approximately 1 mm and the applied load remains within the non-destructive repulsive force regime, the soft probe enables stable, non-destructive electrical contact. In all case studies presented below, the parameters of a commercially available standard soft probe (W wire with *d* = 0.1 mm and *l* = 30 mm) were used. Good contact was consistently established under these conditions, as previously shown [37]. Variations in *l* and *d* for tuning contact force have been previously reported [37].

**Figure 2:**
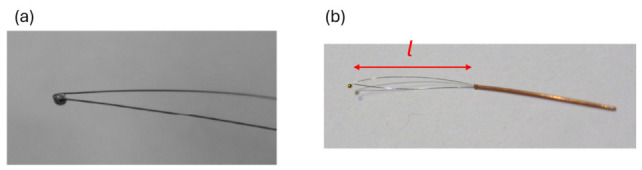
Photographs of soft probe (**a**) and with Cu pipe (**b**) where the wire length *l* is an important parameter for pushing force control (see text).

Figure 2 shows example photographs of the soft probe itself (Figure 2a) and the probe inserted into a Cu tube (Figure 2b). In this configuration, the probe can be electrically connected to a measurement circuit by inserting the Cu tube into a commercially available coaxial cable. The hairpin-shaped structure provides a key mechanical advantage over a single-wire cantilever. In a single-wire configuration, rotational freedom of the cantilever within the coaxial cable can lead to instability in the contact angle between the probe tip and the specimen surface. In contrast, the hairpin geometry fixes the orientation of the hemispherical tip, thereby maintaining a stable and reproducible contact angle. Furthermore, since the applied force can be controlled by adjusting the wire thickness and length, the soft probe can be adapted to a wide variety of specimens with different mechanical properties.

Although various materials can be used for the contacting hemisphere [37] and references therein, Au and Pt-coated Au probes are practical in most cases because of their resistance to surface oxidation. The absence of specimen contamination caused by probe contact has already been confirmed by XPS and optical microscopy [33]. Because the specimens are neither damaged nor contaminated by the probe, electrical contact with the probe is highly repeatable, and the probe can be reused over a long period of time. The lifetime of the probe is mainly limited by improper handling, such as hitting the probe against another object and bending of the Cu pipe.

## 3. Various Advantages of Soft Probe with Examples of Practical Usage

There are many advantages of using soft probes instead of other methods for establishing electrical contact. In this section, we demonstrate these advantages through practical applications of soft probes reported by users.

### 3.1. Prevention of Electrode-Metal Penetration

**Figure 3:**
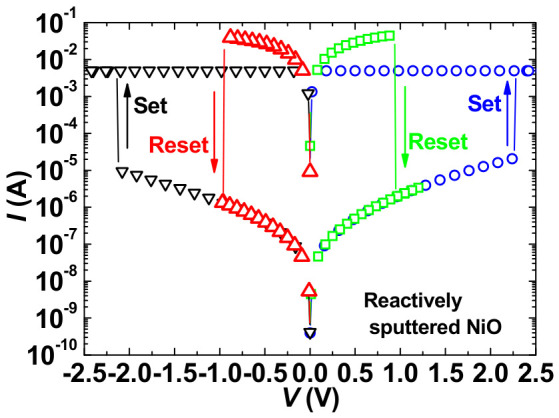
Schematic I−V curves of resistive switching in Pt/NiO/Pt system.

Penetration of evaporated electrode metals is a critical issue, particularly for organic thin films such as self-assembled monolayers (SAMs) and for inorganic films in which grain boundaries play a significant role in their electrical properties. Here, we present an illustrative example using NiO films employed in resistive random-access memory (ReRAM) devices. ReRAM [41] is a type of non-volatile random-access memory (RAM) that operates by modulating the resistance of a dielectric solid-state material. NiO exhibits non-polar resistive switching behavior, as schematically shown in Figure 3. Upon application of a sufficiently high voltage, the resistivity of NiO switches between high- and low-resistance states. In this nonpolar switching mode, both SET (high-to-low) and RESET (low-to-high) transitions occur under both positive and negative voltage polarities.

**Figure 4:**
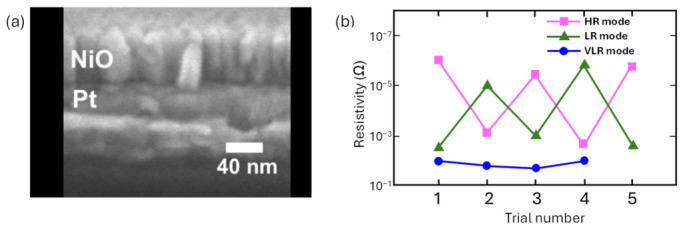
Cross-sectional SEM image of NiO/Pt ReRAM specimen (**a**) and typical resistivity switching behavior in different modes (**b**).

Figure 4a shows a cross-sectional image of the NiO film deposited on a Pt substrate. As shown in the figure, the NiO film exhibits a columnar structure with a thickness of several tens of nanometers, which is a controllable parameter. Figure 4b schematically illustrates the resistivity switching behavior under different operation modes in NiO ReRAM devices. Three modes are identified:

HR mode: the resistivity initially starts high, decreases upon voltage application, and returns to a high state upon subsequent voltage cycling;

LR mode: the resistivity initially starts low, increases upon voltage application, and returns to a low state upon subsequent cycling;

VLR mode: the resistivity remains low regardless of voltage application, indicating the absence of switching behavior.

Repeated switching behavior was observed for more than 200 cycles for both HR and LR modes. The mechanisms underlying these switching modes are discussed in detail in ref. [42].

**Figure 5:**
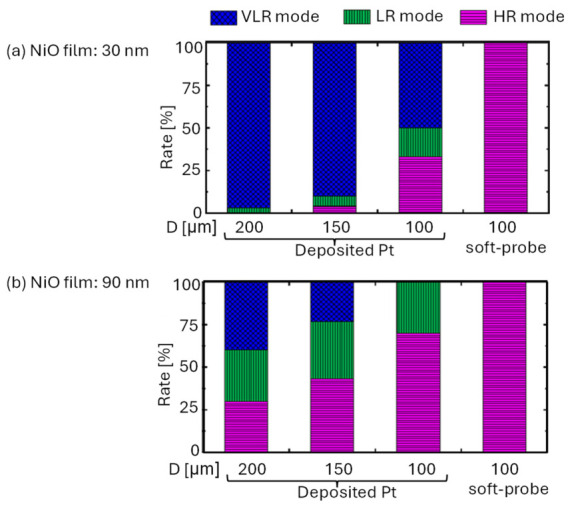
Fraction of three modes in switching with different kinds, and size of electrodes for 30 nm (**a**) and 90 nm (**b**) NiO films.

**Figure 6:**
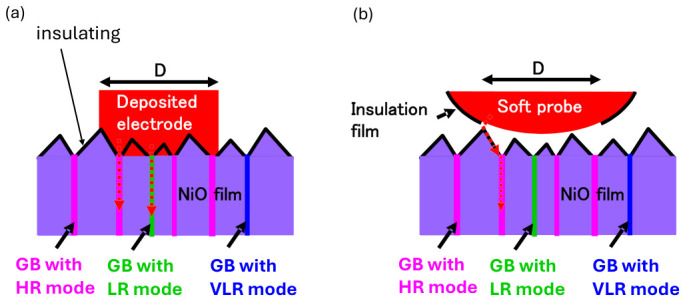
The cross-sectional schematics of the samples with (**a**) deposited electrode and (**b**) soft probe. The red lines show insulating {001} surfaces and the pink, green, and blue lines show HR, LR, and VLR grain surfaces (boundaries), respectively. The red broken arrows show the path through which the electric current flows.

Figure 5 presents the experimental results of resistivity switching for NiO films with thicknesses of 30 nm (Figure 5a) and 90 nm (Figure 5b), comparing deposited Pt electrodes and soft probe contacts. The data in Figure 5 were presented in our prior paper [37]. However, the significance of the results and the advantages of the probe have not been explained previously. Here, a more detailed interpretation of the data is presented. The fractions of the modes were obtained from 30 different specimens with the same structure. When the soft probe was used, 100% HR mode operation was observed for both film thicknesses, indicating uniform and reliable switching behavior. In contrast, when Pt was deposited as a top electrode, the VLR mode was dominant, particularly for the 30 nm-thick film with an electrode diameter (D) of 200 μm, where switching was almost completely suppressed. Although the fraction of HR mode increased with decreasing electrode diameter and increasing film thickness, 100% HR mode was not achieved in any deposited electrode configuration. As discussed in ref. [42], the top surface of the NiO film consists of {001} facets, which are insulating, while the grain boundaries are primarily composed of {11-2} facets, which are conductive. Resistive switching is attributed to a structural transformation at the grain boundaries, where {11-2} conductive regions are converted into insulating {001} regions via displacement of Ni–O atomic pairs. When a soft probe was used, electrical contact was established only at the top insulating {001} surface, resulting in an initially high-resistance state that could subsequently switch to a low-resistance state upon voltage application. Importantly, the probe did not penetrate the grain boundaries and therefore did not directly contact VLR-type conductive pathways, as schematically illustrated in Figure 6b. As a result, only HR mode switching was observed, regardless of film thickness.

In contrast, deposited Pt electrodes make direct contact with both surface regions and grain boundaries. If a VLR-type conductive grain boundary was present within the electrode area, the device exhibited a permanently low resistance with no switching behavior. Similarly, if LR-type grain boundaries were dominant, LR-type switching behavior was observed. Therefore, the dependence of switching modes on electrode diameter D reflects the statistical probability of including different grain-boundary types within the contact area. With increasing film thickness, the probability of forming continuous {11-2} conductive pathways across the entire film thickness decreased, which explains the reduced fraction of the VLR mode in the 90 nm-thick film compared to the 30 nm-thick film. However, even in thicker films, deposited electrodes still exhibited mixed or non-uniform switching behavior due to their direct interaction with grain boundaries (Figure 6a). In contrast, the soft probe enabled selective surface contact without microstructural averaging, resulting in uniform device characteristics.

Furthermore, in this demonstration, the soft probe served as the electrode, eliminating the need for electrode deposition. This offers additional advantages, including reduced fabrication time, energy consumption, and process complexity, in addition to preventing electrode-induced damage and contamination.

### 3.2. Elimination of Electrode Deposition for Ferroelectric Hysteresis Measurements

**Figure 7:**
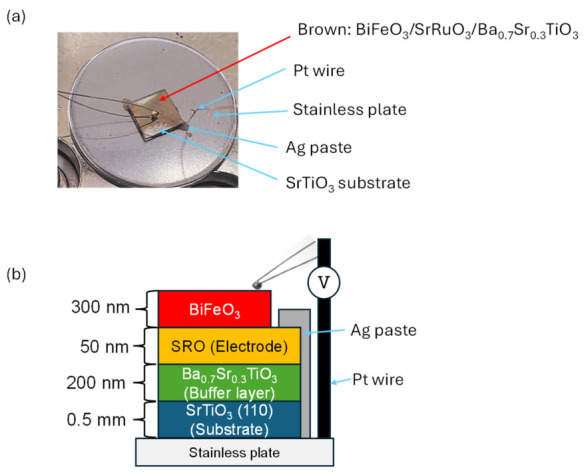
Photo of the sample setup for the measurement using a soft probe (**a**), and the schematically shown cross-section of the setup (**b**).

Using a soft-probe as an electrode provides several advantages: (1) elimination of the electrode deposition process, thereby reducing time, material consumption, and labor; (2) avoidance of penetration by evaporated electrode metals into the specimen, as shown in Section 3.1; (3) prevention of contamination of the specimen by electrode metals [33], allowing subsequent measurements on the same sample; and (4) formation of sufficiently good electrical contact to observe sharp hysteresis in dielectric materials. Since the first three advantages have already been demonstrated in previous studies or Section 3.1, the fourth advantage is examined here through a practical application to ferroelectric films. Figure 7a shows an example photograph of the measurement setup for a BiFeO_3_ epitaxial film using a soft probe with a Au tip; the structure is schematically illustrated in Figure 7b. These films are epitaxial, and therefore electrode-metal penetration is not a significant issue due to the low density of grain boundaries, as discussed in Section 3.1. Nevertheless, the use of a soft probe as an electrode provides an additional advantage, as described below.

**Figure 8:**
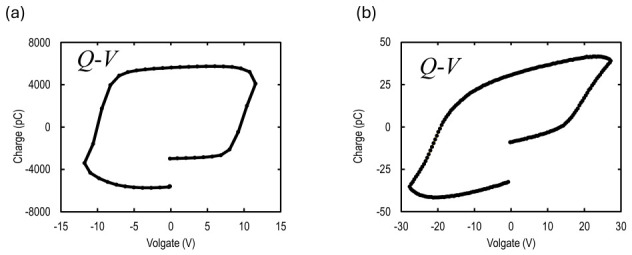
Q-V curve measured with (**a**) Au deposited film with the area of 0.0156 mm^2^ and (**b**) Au soft probe.

The polarization characteristics of the epitaxial BiFeO_3_ film measured using a soft probe are shown in Figure 8b, while the results obtained using a conventional deposited electrode are shown in Figure 8a. Here, charge is plotted rather than polarization for comparison because the contact area with the soft probe is unknown. For comparison of the curve shapes, normalization by the contact area is not essential. The Q-V curve in Figure 8b exhibits a clear hysteresis loop with polarization reversal, similar to that observed in Figure 8a, indicating that the soft probe can function as a deposition-free electrode. Furthermore, the hysteresis loop in Figure 8b shows sharper transitions at the top-right and bottom-left regions, corresponding to the reversal of the applied electric field, compared with the curve in Figure 8a. The sharper hysteresis is attributed to reduced leakage current [43]. A low leakage current associated with a small contact area is well known for this type of materials [43]. A small contact area significantly increases the probability of avoiding conductive regions, in a manner analogous to the mechanism discussed in Section 3.1, although this effect is not related to electrode-metal penetration. Therefore, the low leakage current observed with the soft probe is likely attributed to its small contact area. This interpretation is supported by the fact that the measured charge in Figure 8a is approximately two orders of magnitude larger than that in Figure 8b. The contact area in Figure 8a is 0.0156 mm^2^, whereas the contact area of the soft probe is estimated to be approximately two orders of magnitude smaller, based on the discussion in Section 2. Therefore, the use of the soft probe is superior to that of deposited electrodes in several respects, including the elimination of the deposition process, avoidance of contamination, and reduction in leakage-current pathways beneath the contact area. In this example, voltage application with negligible current flow was achieved using the soft probe. A similar clear hysteresis loop was also observed on another ferroelectric film BaTiO_3_ [44].

### 3.3. Applicability to Small and Fragile Materials

Soft probes can be applied to small and mechanically fragile specimens, for which conventional electrical measurement techniques are not suitable. In many cases, individual specimens are too small to fabricate standard electrodes, or the materials themselves are structurally vulnerable to external mechanical or thermal stress. Here, we demonstrate an example of electrical characterization of a metal–organic framework (MOF) using a soft probe. MOFs are a class of coordination polymers consisting of metal ions or metal-containing secondary building units (SBUs), coordinated to organic ligands to form one-, two-, or three-dimensional structures that are usually porous [45]. These materials are typically highly fragile and often exist as powdery or micrometer-sized crystals [46,47]. It is widely recognized that establishing electrical contact with MOFs is very difficult [15]. The conventional approach of metal electrode deposition can lead to penetration of metals into interparticle gaps, resulting in electrical short circuits. Alternatively, powder specimens are often compressed into pellets for measurement; however, this process collapses the intrinsic porous structure, thereby altering the material’s properties and effectively destroying the MOF characteristics. In contrast, the soft probe enables direct electrical contact without applying excessive mechanical stress, owing to its compliant spring-based structure. This allows electrical measurements to be performed without collapsing the pore structure of the MOF.

As a representative example, we demonstrate successful electrical measurements of HKUST-1 ((Cu_3_(BTC)_2_); BTC = benzene-1, 3, 5-tricarboxylic acid) [48]. The typical crystal size of this MOF is 10–20 μm. Although electrical measurements of the same specimens have been shown in our prior paper [37], detailed explanations of the specimen and measurement conditions were missing from the paper. Here, the figures have been reexamined and revised based on the previously reported data, and explanations of the experimental procedure have been added.

**Figure 9:**
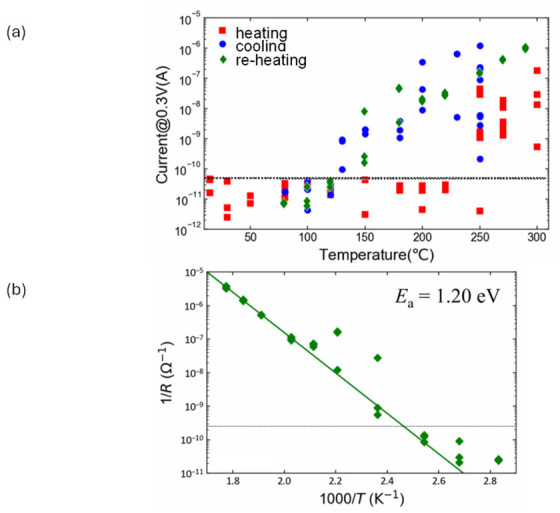
Temperature dependence of current at 0.3 V (**a**) and Arrhenius plot of resistance (**b**) of MOF. The dashed lines both in (**a**,**b**) show the measurement limits, which means values lower than the lines are within noise level.

Figure 9a shows the temperature dependence of the electrical current measured at an applied voltage of 0.3 V, which is proportional to the inverse of the resistance. During the first heating cycle, the current remains low up to approximately 250 °C, above which it increases significantly. During subsequent cooling, the current gradually decreases and stabilizes below approximately 100 °C. In the second heating cycle, the temperature dependence of the current closely follows that observed during cooling. This difference between the first and second heating cycles can be attributed to the presence of adsorbed water molecules within the MOF pores in the initial state. Upon heating, these water molecules desorb and evaporate [48], resulting in a reproducible intrinsic electrical response during subsequent thermal cycles.

Figure 9b shows the activation energy for electrical conduction obtained during the reheating process. Because the soft probe maintained stable contact with the same microcrystals throughout the temperature variation, consistent and reliable data were obtained. Furthermore, the contact between the MOF and the soft probe was confirmed to be ohmic, indicating that the measured activation energy reflects intrinsic transport mechanisms, such as carrier hopping within the MOF structure. There was no difference in the XRD patterns before and after electrical measurements [37], confirming the absence of structural damage caused by probe contact. In this example, both voltage application and current measurement were performed through the soft probe, demonstrating its capability for reliable electrical characterization of fragile, microscale materials without structural degradation.

### 3.4. Vibration- and Thermal Stress-Resistant Measurements

When a novel material is discovered, measurement of its fundamental physical properties, including electrical conductivity and dielectric response, is essential for both scientific understanding and device applications. For measurements of the temperature dependence of electrical properties at low temperatures, evacuation of the measurement system is generally required to avoid moisture condensation. In such measurement environments, mechanical vibration is unavoidable, as vacuum systems typically use pumps that generate vibration. In some cases, low temperatures are achieved using cryogenic systems based on gas compression and expansion, which also introduce additional vibration. To establish electrical contact under these conditions, thin Au wires connected to electrical feedthroughs are commonly attached to specimens using conductive pastes, such as Ag or carbon-based adhesives. However, this conventional approach has significant limitations. Because the mechanical response of the wire differs from those of the specimen and its holder, their vibrational motions are not synchronized. As a result, mechanical stress is generated at the interface between the wire and the specimen, often leading to fracture or detachment of the conductive paste and consequently to loss of electrical contact during measurement.

Another critical issue arises from thermal stress during cooling. Due to the mismatch in thermal expansion coefficients between the specimen and the conductive paste, thermal contraction induces mechanical stress at the interface, which can also cause failure of electrical contact.

It is well known in the field of low-temperature physics that the mechanical and thermal stress described above often results in the breakdown of the electrical connection between the Au wire and the paste. The connection may be restored intermittently; however, it remains unstable or incomplete, resulting in unstable signals and high noise levels.

The soft probe provides an effective solution to these problems. Because of its spring-like mechanical compliance, the probe can absorb both vibrational and thermal stresses, maintaining stable contact with the specimen. The hairpin-shaped structure applies a controlled force through elastic deformation, allowing the probe tip to remain in contact with the surface without rigid mechanical constraint. In addition to these mechanical advantages, the soft probe also offers non-contaminating electrical contact, enabling the same specimen to be used for multiple measurements.

**Figure 10:**
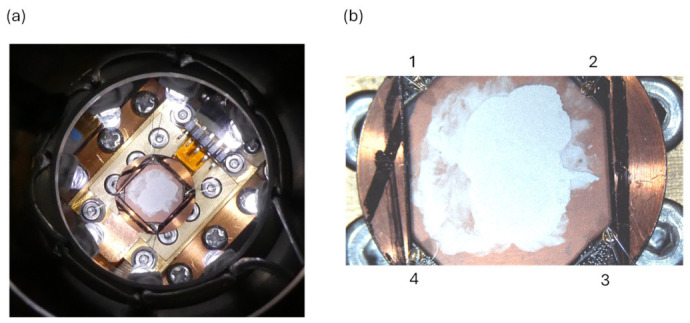
Photos of specimen setting in the vacuum chamber (**a**) and expanded view around the specimen with probe number 1–4 indicated (**b**).

**Figure 11:**
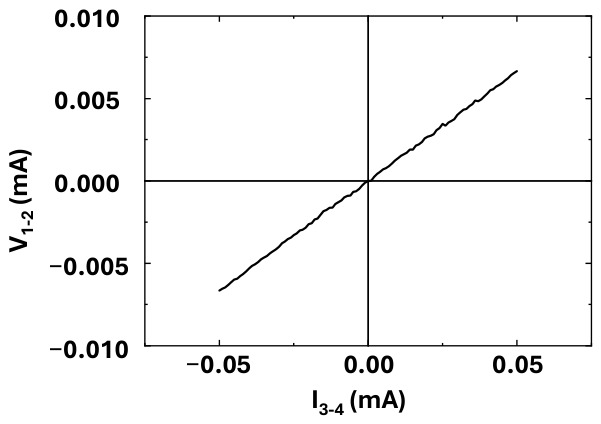
Sheet resistance measurement of single-layer graphene using current flow between electrode 3 and 4 under voltage application between electrode 1 and 2.

As a demonstration, low-temperature electrical measurements of a graphene sample under a magnetic field are presented [49,50]. The specimen, consisting of single-layer graphene on a sapphire substrate, was placed in a vacuum chamber connected to a turbomolecular pump and a rotary pump. The chamber was designed to be inserted into a magnet system for Hall-effect measurements. Figure 10a shows a top view of the specimen and probe configuration inside the vacuum chamber, while Figure 10b provides a magnified view of the contact region. The large white area corresponds to silver paste beneath the sapphire substrate, and four soft probes are positioned on the triangular Au electrodes deposited at the corners of the graphene layer, which covers the entire surface of the sapphire substrate. The specimen temperature was reduced using a compressor-based cooling system. Despite the presence of continuous vibration from the vacuum pumps and compressor, as well as thermal stress induced by temperature change, stable electrical measurements were successfully achieved at 32 K, as shown in Figure 11. Furthermore, Hall- effect measurements were successfully performed, demonstrating that the soft probe enables reliable electrical characterization even under mechanically and thermally demanding conditions.

## 4. Discussion

The results presented in Section 3 demonstrate that the advantages of the soft probe are not isolated phenomena specific to individual material systems, but instead originate from a unified physical mechanism. The essential feature of the soft probe is its ability to realize stable electrical contact in the repulsive-force regime without the need for active feedback control. This characteristic fundamentally differentiates the soft probe from conventional electrical contact methods, such as deposited electrodes and rigid metallic needle probes.

One of the most significant implications of this mechanism is the ability to avoid direct interaction with microstructural heterogeneities. In the NiO ReRAM example, the soft probe selectively contacted the insulating {001} surface without penetrating grain boundaries. As a result, variability in switching behavior was eliminated, and uniform HR-mode operation was achieved. In contrast, deposited electrodes inevitably average over heterogeneous microstructures, leading to statistical variations in device characteristics. This indicates that the soft probe can effectively define the electrically active contact region at the microscale.

Another important advantage is the elimination of electrode deposition. Conventional electrode fabrication introduces several issues, including contamination, thermal damage during deposition, and structural modification of specimens. By contrast, the soft probe enables direct electrical contact without altering the material. The ferroelectric measurements further demonstrate that this approach not only preserves the intrinsic properties of the specimen but can also improve measurement quality. The sharper hysteresis observed with the soft probe suggests a reduction in leakage current, which can be attributed to the smaller effective contact area and the reduced probability of contacting conductive regions.

The applicability to fragile and microscale materials further highlights the versatility of the soft probe. In the case of MOF crystals, conventional methods such as metal deposition or pelletization fundamentally alter the structure of the material. The soft probe, owing to its mechanically compliant design, enables electrical measurements while preserving the intrinsic porous structure. This is particularly important for emerging materials in which structural integrity is directly linked to functional properties.

In addition, the soft probe provides a robust solution for measurements under mechanical or thermal disturbances. The spring-like structure absorbs both vibrational and thermal stresses, maintaining stable electrical contact even under vacuum, low-temperature, and magnetic-field environments. This capability addresses a long-standing challenge in experimental setups, where conventional wiring and conductive pastes can fail.

Although the examples above do not experimentally demonstrate this capability, the soft probe itself can be integrated with mapping capability for electrical measurements and with simultaneous multipoint measurements [34].

From a broader perspective, the soft probe can be regarded as a platform technology for electrical measurements, rather than a simple replacement for conventional probes. By combining mechanical compliance with electrical functionality, it enables new measurement regimes that are difficult or impossible to achieve using conventional approaches. These include selective contact with specific microstructural regions, reliable measurements of fragile or small-scale specimens, and stable operation under extreme environments.

Despite these advantages, several limitations should be considered. The contact area and applied force are determined by probe geometry and manual operation, which may introduce variability if not properly controlled. In addition, applications requiring well-defined electrode geometries or high-frequency measurements may require further optimization of probe design. Future work should focus on the quantitative control of contact mechanics, the integration with automated positioning systems, and extension to high-frequency or high-throughput measurement platforms.

## 5. Conclusions

In this study, we have demonstrated that the soft probe, originally developed for non-destructive electrical contact, provides a wide range of additional advantages arising from its mechanically compliant design. By enabling stable electrical contact in the repulsive-force regime without active force control, the soft probe allows reliable measurements while minimizing mechanical damage and contamination.

Through representative case studies, we have shown that the soft probe eliminates the need for electrode deposition, thereby avoiding problems such as metal penetration, thermal damage during deposition, and contamination. In NiO ReRAM measurements, the soft probe enabled uniform switching behavior by preventing direct contact with conductive grain boundaries. In ferroelectric measurements, it provided improved hysteresis characteristics through reduced leakage current. Furthermore, its successful application to MOF crystals demonstrated its capability to preserve the intrinsic structures of fragile materials during measurement.

In addition, the soft probe exhibits excellent robustness against external disturbances, including vibration and thermal stress. Its spring-like structure absorbs mechanical stress arising from vibrational mismatch and differences in thermal expansion, enabling stable electrical measurements under demanding conditions such as vacuum, low temperatures, and magnetic fields.

These results indicate that the soft probe is not merely an alternative to conventional electrical contact methods, but also a versatile enabling technology that expands the scope of electrical measurements. Its adaptability to diverse material systems—from thin films and microstructured materials to fragile and porous systems—makes it particularly valuable for emerging materials research.

Overall, the soft probe provides a simple, reliable, and contamination-free means of establishing electrical contact and is expected to contribute significantly to both fundamental studies and practical device evaluations across a wide range of material systems.

## Data Availability

The original contributions presented in this study are included in the article. Further inquiries can be directed to the corresponding author.
